# Annotations

**Published:** 1907-03-30

**Authors:** 


### ANNOTATIONS.

### A National Anthropometric Survey.

The deputation which waited on the Prime Minister in the early part of March to urge the institution of a national anthropometric survey hardly succeeded in their direct object, but their intervention will doubtless prove of educational value. Their proposal, apart from other merits, has this supreme virtue, that it is an endeavour to get at the actual facts of the case. We have had many statements and opinions and views regarding the physical deterioration of the British people, and many suggested checks for the alleged downward movement. But with these there has not been any considerable and authoritative body of facts collected over a large area. Hence there have arisen contradictions which, perhaps, have not had any better foundation than the original propositions. And altogether, the question, which is obviously one of great national importance, has been left in a highly unsatisfactory and indefinite position. An authoritative anthropometric survey would not all at once relieve this position. But if steadily pursued it would in course of time mean the accumulation of a body of facts presented in terms of exact measurement, and open to comparative judgment and estimate. Such a survey is the only arrangement by which we can study the changes in the condition of the people at large as a guide to action in administration and legislation. At present, here, as in other directions of national economy, we fumble and grope our way without any real and definite knowledge of the facts with which we are dealing. The estimated annual cost of the proposed survey is about £4,500. But the money is not the only difficulty. Public opinion must be educated before British parents will allow their children in public or other schools to be made the subjects of exact observations for scientific record. The distrust of the scientist is an acute conviction of the average Briton.

### Free Municipal Antitoxin.

The Northern Medical Society of Glasgow has recently presented an interesting memorial urging upon the Municipality the desirability of establishing serum depôts from which poor patients may obtain supplies of anti-diphtheritic serum free of charge. The signatories point out that prompt and energetic injection of the antitoxin is now considered to be of paramount importance in successfully combating the disease. Other health authorities have already decided to establish such free depôts. Chicago started one in 1895, when its death-rate from diphtheria was 35 per cent., and suceeded in reducing this appalling mortality to 6 per cent. In the same year New York established a free serum depôt. Since then depôts on similar lines have been established in New Zealand, Brazil, Pennsylvania, Guernsey, and, more recently, in Birmingham. The cogent arguments with which the signatories drive home their appeal need no commendation to medical men. Every physician knows the difficulty that faces him in dealing with some cases of diphtheria, especially in poor patients. He knows that it is a hardship, at times a virtual impossibility, for the parents of the poor child to purchase the antitoxin; yet he cannot, or dare not, drive the patient into a hospital, where the remedy may be obtained free of charge. No doubt in time all our large cities will follow Birmingham's example and establish such depôts as the memorialists of Glasgow pray for. In the interests of the community the expense entailed will be more than justified.

### St. Andrew v. St. John.

The Order of St. John of Jerusalem recently petitioned the Privy Council for a supplementary charter to enable it to establish Priories in Scotland, or, indeed in any part of the empire, its activities, so far as the Ambulance Association is concerned, having been confined hitherto to England and Wales. Scotland already has the St. Andrew's Ambulance Association, which has done, and is doing, excellent and appreciated service across the border, and this association, and Scotsmen too, naturally regard the intention of the sister body to establish opposition priories in so well covered a sphere of work as unjustifiable and unnecessary. A counter-petition has therefore been lodged praying that the prayer of the St. John's Ambulance Association be not granted, but St. Andrew's offers to act as ambulance branch of the sister order so long as its own independence under its original Royal Charter is guaranteed. In taking this step, the St. Andrew's Association has roused its president, Lord Breadalbane, who is a member of the executive of the Order of St. John, and who feels compelled to resign his connection with the Scottish Association in consequence of the latter's opposition to the original petition. Lord Breadalbane suggests that in the event of the St. Andrew's petition proving successful—a contingency which he considers "most unlikely"—it will merely prevent the St. John's Order from establishing a priory, but not from starting ambulance classes, and will in no way strengthen friendly relations between the two Orders. This is certainly a very regrettable utterance on Lord Breadalbane's part as the retiring president of St. Andrew's Priory. The St. Andrew's Association has achieved so much success and is doing such excellent work that there is no justification for interfering with its labours. It has acted in a charitable and sisterly spirit in proffering its services to the St. John's Association under a guarantee of independence, and the latter association, in somewhat rudely rejecting that offer, has shown a spirit which we regret to observe in any charitable body. Everyone who impartially faces the facts will admit that the St. John's Association is acting indiscreetly in attempting to force itself upon Scotland, which already possesses so excellent an ambulance association as that of St. Andrew's. Under the circumstances we consider the rejection of the original petition by no means so unlikely a contingency as Lord Breadalbane seems to think. Such rejection would be in accord with the wishes of the people of Scotland, who ought to be left free to manage their own ambulance business in their own way.

